# Dietary B group vitamin intake and the bladder cancer risk: a pooled analysis of prospective cohort studies

**DOI:** 10.1007/s00394-022-02805-2

**Published:** 2022-02-07

**Authors:** Iris W. A. Boot, Anke Wesselius, Evan Y. W. Yu, Maree Brinkman, Piet van den Brandt, Eric J. Grant, Emily White, Elisabete Weiderpass, Pietro Ferrari, Matthias B. Schulze, Bas Bueno-de-Mesquita, Maria Jose-Sanchez, Bjorn Gylling, Maurice P. Zeegers

**Affiliations:** 1 Department of Complex Genetics and Epidemiology, School of Nutrition and Translational Research in Metabolism, Maastricht University, Universiteitssingel 40 (Room C5.570), 6229 ER Maastricht, The Netherlands; 2 Department of Clinical Studies and Nutritional Epidemiology, Nutrition Biomed Research Institute, Melbourne, Australia; 3 Cancer Epidemiology Division, Cancer Council Victoria, Melbourne, VIC Australia; 4 Department of Epidemiology, Schools for Oncology and Developmental Biology and Public Health and Primary Care, Maastricht University Medical Centre, Maastricht, The Netherlands; 5 Department of Epidemiology Radiation Effects Research Foundation, Hiroshima, Japan; 6 Fred Hutchinson Cancer Research Center, Seattle, WA USA; 7 International Agency for Research on Cancer World Health Organization, Lyon, France; 8 Department of Molecular Epidemiology, German Institute of Human Nutrition Potsdam-Rehbruecke, Nuthetal, Germany; 9 Institute of Nutritional Science, University of Potsdam, Nuthetal, Germany; 10 Department for Determinants of Chronic Diseases, National Institute for Public Health and the Environment, Bilthoven, The Netherlands; 11 Escuela Andaluza de Salud Publia, Granada, Spain; 12 Instituto de Investigación Biosanitaria, Granada, Spain; 13 Centro de Investigación Biomédica en Red de Epidemiología y Salud Pública, Madrid, Spain; 14 Department of Preventive Medicine and Public Health, University of Granada, Granada, Spain; 15 Department of Medical Biosciences, Pathology, Umeå University, Umeå, Sweden; 16 CAPHRI School for Public Health and Primary Care, Maastricht University, Maastricht, The Netherlands; 17 School of Cancer Sciences, University of Birmingham, Birmingham, UK

**Keywords:** Nutritional oncology, Bladder cancer, Pooled cohort analysis, B group vitamins

## Abstract

**Purpose:**

Diet may play an essential role in the aetiology of bladder cancer (BC). The B group complex vitamins involve diverse biological functions that could be influential in cancer prevention. The aim of the present study was to investigate the association between various components of the B group vitamin complex and BC risk.

**Methods:**

Dietary data were pooled from four cohort studies. Food item intake was converted to daily intakes of B group vitamins and pooled multivariate hazard ratios (HRs), with corresponding 95% confidence intervals (CIs), were obtained using Cox-regression models. Dose–response relationships were examined using a nonparametric test for trend.

**Results:**

In total, 2915 BC cases and 530,012 non-cases were included in the analyses. The present study showed an increased BC risk for moderate intake of vitamin B1 (HR_B1_: 1.13, 95% CI: 1.00–1.20). In men, moderate intake of the vitamins B1, B2, energy-related vitamins and high intake of vitamin B1 were associated with an increased BC risk (HR (95% CI): 1.13 (1.02–1.26), 1.14 (1.02–1.26), 1.13 (1.02–1.26; 1.13 (1.02–1.26), respectively). In women, high intake of all vitamins and vitamin combinations, except for the entire complex, showed an inverse association (HR (95% CI): 0.80 (0.67–0.97), 0.83 (0.70–1.00); 0.77 (0.63–0.93), 0.73 (0.61–0.88), 0.82 (0.68–0.99), 0.79 (0.66–0.95), 0.80 (0.66–0.96), 0.74 (0.62–0.89), 0.76 (0.63–0.92), respectively). Dose–response analyses showed an increased BC risk for higher intake of vitamin B1 and B12.

**Conclusion:**

Our findings highlight the importance of future research on the food sources of B group vitamins in the context of the overall and sex-stratified diet.

**Supplementary Information:**

The online version contains supplementary material available at 10.1007/s00394-022-02805-2.

## Introduction

With an estimated 549,393 new cases and 199,922 deaths in 2018, bladder cancer (BC) is the tenth most common cancer worldwide [1]. More than half of all BC cases occur in higher income countries, with the highest incidence rates in North America and Europe and the lowest in Africa. Due to high recurrence rates, BC is an expensive malignancy to treat from diagnosis to death, with estimated costs ranging from USD 89,287 to USD 202,203 per patient [2]. In fact, bladder cancer is the most expensive malignancy to treat of all cancers [3]. Therefore, BC is an important public health problem.

BC is a complex disease not only influenced by genetic predisposition, but also lifestyle, environmental, and occupational exposures could potentially play an important role in the development of this disease [4]. The more established risk factors associated with the development of BC include smoking and deleterious occupational exposure [5, 6] and male gender [5, 7]. Since the bladder is an important excretion organ through which dietary metabolites are filtered, diet may play an essential role in the development of BC. According to the United States National Cancer institute, one third of all BC cases could have been prevented by adherence to dietary recommendations, hence the salient need to investigate potential associations between foods, nutrients and BC [8].

Previous epidemiological research on diet and BC reported that high amount of fluid, fruit, vegetable, yogurt, whole grain and dietary fiber intake were associated with a reduced risk of BC [9–11], while higher intake of barbecued meat, pork, and total fat may increase BC risk [8]. At last, organ meat consumption has also been associated with BC development [12].

Although these findings for individual food items lead to useful dietary recommendations, it remains unclear what nutrients or bioactive compounds are responsible for the observed effects on BC risk [13].

The B group complex vitamins are found in a variety of foods, including meat, whole grains, and are especially in rich supply in fruits and vegetables [14]. The B group complex vitamins include (1) thiamine (B1), (2) riboflavin (B2) and (3) niacin (B3), which mainly play a role in energy metabolism [15–17], (4) pyridoxine (B6), which reduces oxidative stress (as does vitamin B2), thereby preventing DNA damage [18, 19], (5) folate (B9), and (6) cyanocobalamin (B12), which play a significant role in protecting and maintaining the stability of the human genome by the maintenance of one-carbon metabolism (i.e. a set of interconnected biochemical pathways driven by both vitamin B9 and B12 to generate methyl groups for DNA methylation [20]), thereby possessing the potential to lower the chance of a neoplastic events [14, 21]. However, despite the biological plausibility that these B group vitamins play a role in cancer prevention, epidemiological evidence on the effect of these B group vitamin compounds on BC development is lacking or inconclusive [4, 14, 22]. Inverse associations with BC risk have only been reported for high intake of the vitamins B9, B12 and B6 [4, 23, 24]. A meta-analysis, however, concluded that the evidence for this protective effect is limited [14].

This lack of evidence could be due to the small sample sizes of previous studies and their consequent lack of statistical power to detect associations. The present study, therefore, aims to provide a more precise quantitative estimate for the associations between the various components of the B group complex vitamins and BC risk by pooling data from four cohort studies.

## Materials and methods

### Study population

Data were derived from the *BL*adder cancer *E*pidemiology and *N*utritional *D*eterminants study (BLEND): a large international consortium on dietary factors and BC risk, compromising a total of 11,000 cases and over 680,000 non-cases aged between 18 and 100 years from different countries in Europe, America, Asia and Australia [25]. Currently, BLEND consists of 19 case–control and seven cohort studies.

The present study pooled data from the BLEND cohort studies only. Four cohort studies, including a total of 2915 cases and 530,012 non-cases, had sufficient information on dietary B group vitamin intake to be eligible for inclusion in our analyses. Informed consent was obtained from all individual participants included in each study. Included studies were the Netherlands Cohort Study on diet and cancer (NLCS) [26], the Radiation Effects Research Foundation (RERF) atomic bomb survivors study [27], the VITamins And Lifestyle study (VITAL) [28] and the European Prospective Investigation into Cancer and nutrition (EPIC) [29, 30] (Table 1).

**Table 1:** Baseline characteristics of the four included cohort studies

	NLCS		RERF		VITAL		EPIC		Overall	
	No	%	No	%	No	%	No	%	No	%
Country	The Netherlands		Japan		USA		Europe			
Recruitment period	1986–2003		1950–2000		2000–2008		1993–2006			
Food items (n)	150		102		126		260			
Mean follow-up, years (± SD)	14.06 (± 4.81)		27.78 (± 12.74)		6.74 (± 1.50)		11.04 (± 2.81)		11.20 (± 4.50)	
Subject status										
Total	5247	100	6582	100	69,489	100	451,609	100	532,927	100
Cases	877	16.71	42	0.64	346	0.50	1,650	0.37	2915	0.55
*Men*	734	83.69	32	76.19	269	77.75	1,154	69.94	2,189	75.09
*Women*	143	16.31	10	23.81	77	22.25	496	30.06	726	24.91
Non-cases	4370	83.29	6540	99.36	69,143	99.50	449,959	99.63	530,012	99.45
*Men*	2134	48.83	2475	37.84	33,470	48.41	131,503	29.23	169,582	32.00
*Women*	2236	51.17	4065	62.16	35,673	51.59	318,456	70.77	360,430	68.00
Sex										
Men	2868	54.66	2507	38.09	33,739	48.55	132,657	29.37	171,771	32.23
Women	2379	45.34	4075	61.91	35,750	51.45	318,952	70.63	361,156	67.77
Age (years)										
< 50										
Cases			18	42.86	2	0.58	200	12.12	220	7.55
Non-cases			3703	56.62	1812	2.62	191,060	42.46	196,575	37.09
50–59										
Cases	195	22.23	10	23.81	71	20.52	617	37.39	893	30.63
Non-cases	1355	31.01	1179	18.03	30,845	44.61	160,674	35.71	194,053	36.61
60–69										
Cases	631	71.95	10	23.81	132	38.15	725	43.94	1498	51.39
Non-cases	2781	63.64	1198	18.32	23,862	34.51	88,032	19.56	115,873	21.86
> = 70										
Cases	51	5.82	4	9.52	141	40.75	108	6.55	304	10.43
Non-cases	234	5.35	460	7.03	12,624	18.26	10,193	2.27	23,511	4.44
TNM stage										
MIBC	413	51.69			110	32.74	133	21.63	656	37.49
*Male*	333	80.63			91	82.73	98	73.68	522	79.57
*Female*	80	19.37			19	17.27	35	26.32	134	20.43
NMIBC	386	48.31			226	67.26	482	78.37	1094	62.51
*Male*	335	86.79			169	74.78	313	64.94	817	74.68
*Female*	51	13.21			57	25.22	169	35.06	277	25.32
Smoking status										
Cases										
Never	117	13.34	7	16.67	85	24.57	349	21.15	558	19.14
Current light	63	7.18					164	9.94	227	7.79
Current heavy	287	32.73					433	26.24	720	24.70
Current unknown	36	4.10	28	66.67	53	15.32	92	5.58	209	7.17
Former light	127	14.48							127	4.36
Former heavy	224	25.54							224	7.68
Former unknown	23	2.62	7	16.67	208	60.12	612	37.09	850	29.16
Non-cases										
Never	1581	36.18	3579	54.72	32,982	47.70	223,234	49.61	261,376	49.32
Current light	353	8.08					48,134	10.70	48,487	9.15
Current heavy	765	17.51					43,435	9.65	44,200	8.34
Current unknown	115	2.63	2580	39.45	5621	8.13	10,546	2.3	18,862	3.56
Former light	820	18.76							820	0.15
Former heavy	647	14.81							647	0.12
Former unknown	89	2.04	381	5.83	30,540	44.17	124,610	27.69	155,620	29.36
Thiamine (B1)^a^										
Mean (± SD)										
Cases	2.80 (± 0.63)		0.12 (± 0.08)		0.42 (±0.11)		1.33 (± 0.45)		1.65 (± 0.35)	
Non-cases	2.65 (± 0.26)		0.12 (± 0.01)		0.40 (±0.01)		1.10 (± 0.02)		1.01 (± 0.01)	
*t* (*p*)	2.40 (0.02)		0.70 (0.48)		1.87 (0.06)		9.12 (< 0.001)		34.11 (< 0.001)	
Riboflavin (B2) ^a^										
Mean (± SD)							1.42 (± 0.09)			3.63 (± 0.13)
Cases	9.17 (± 0.32)		0.13 (± 0.01)		0.56 (± 0.02)		0.98 (± 0.003)			0.97 (± 0.003)
Non-cases	8.60 (± 0.13)		0.12 (± 0.001)		0.53 (± 0.002)		9.60 (< 0.001)			68.32 (< 0.001)
*t* (*p*)	1.71 (0.09)		0.45 (0.65)		1.21 (0.23)					
Niacin (B3) ^a^										
Mean (± SD)										
Cases	22.23 (± 0.29)		0.73 (± 0.06)		6.87 (±0.18)		16.45 (±0.64)		16.83 (±0.38)	
Non-cases	20.89 (± 0.12)		0.71 (± 0.01)		6.54 (±0.01)		13.05 (±0.02)		12.11 (±0.02)	
*t* (*p*)	4.41 (< 0.001)		0.33 (0.74)		1.86 (0.06)		9.98 (<0.001)		19.38 (<0.001)	
Pyridoxine (B6) ^a^										
Mean (± SD)										
Cases	2.73 (± 0.07)		0.04 (± 0.003)		0.61 (± 0.02)		2.04 (± 0.09)		2.05 (± 0.06)	
Non-cases	2.60 (± 0.03)		0.03 (± 0.00002)		0.65 (± 0.001)		1.66 (± 0.003)		1.51 (± 0.003)	
*t* (*p*)	1.96 (0.05)		1.19 (0.23)		− 2.02 (0.04)		7.58 (< 0.001)		14.96 (< 0.001)	
Folate (B9) ^b^										
Mean (± SD)										
Cases	512.85 (± 7.73)				97.55 (± 2.70)		199.39 (± 2.87)		282.81 (± 4.10)	
Non-cases	488.49 (± 3.29)				97.54 (± 0.20)		196.18 (± 0.15)		185.59 (± 0.15)	
*t* (*p*)	3.00 (0.003)				0.005 (1.00)		1.29 (0.20)		48.27 (< 0.001)	
Cyanocobalamin (B12)^b^										
Mean (± SD)										
Cases	9.64 (± 0.44)		0.03 (± 0.003)		3.92 (± 0.20)		3.10 (± 0.09)		5.12 (± 0.15)	
Non-cases	9.67 (± 0.18)		0.03 (± 0.0003)		4.26 (± 0.01)		5.32 (± 0.01)		5.15 (± 0.01)	
*t* (*p*)	− 0.06 (0.95)		0.14 (0.89)		− 1.68 (0.09)		− 14.10 (< 0.001)		− 0.28 (0.78)	

*NLCS* Netherlands Cohort Study on diet and cancer, *RERF* the Radiation Effects Research Foundation, *VITAL* the VITamins And Lifestyle study, *EPIC* European Prospective Investigation into Cancer and nutrition, *TNM* Classification of Malignant tumors, *SD* standard deviation

^a^Nutrient values measured per day in milligrams

^b^Nutrient values measured per day in micrograms

### Data collection and coding

Details on the methodology of the BLEND consortium have been described elsewhere [25]. All included studies used a self-administered or trained interviewer administered food frequency questionnaire (FFQ) that was validated on either food groups [28, 30–33], and/or energy intake [33, 34]. The period of recalling the dietary intake and the method used to validate the intake differed per study. In brief, (a) in the NLCS participants were asked to report on their dietary intake during the preceding year before study enrolment. This method was validated by a 5-year reproducibility test [35]; (b) the Vital study used a time reference for all dietary questions of “in the last 3 months” and used 24-h dietary recalls and a 4-day food record to validate the dietary intake of the participants [28]; (c) studies included in the EPIC study used either a 7-day food consumption diary or 24-h dietary recalls to validate the reported dietary intake of the participants during the preceding year [29]; (d) the RERF also used 24-h recalls to assess the bias and precision of their FFQ in which a 1 year time reference was used [32]. The collected dietary data were harmonized and categorized using the hierarchical Eurocode 2 food coding system (ECC) developed by the European Union, besides, weekly, monthly or yearly intake were converted to weekly food intake.

As a second step, all recoded food items were converted into nutrients using the United States Department of Agriculture (USDA) food composition database [36]. This database has been validated for B group vitamins [37]. For this, we chose the nutrient content per 100 g of generic food items where possible. Raw products were preferred over cooked/boiled for fruits, whereas for meat, fish, vegetables (except for salad vegetables—see below) and pulses roasted/cooked/boiled/grilled was preferred over raw. For example, for “Whitefish” we chose "Fish, whitefish, mixed species, cooked, dry heat" (code 15223) and for “Chicken breast” we chose “Chicken, broilers or fryers, breast, skinless, boneless, meat only, cooked, grilled” (code 05747). The nutrient content for endive, lettuce, fennel, chicory, garlic, chives, radish, cucumber, avocado, morel, and cress was selected as raw, for tomatoes the raw and cooked values were averaged, and for olives “canned” was selected.

Two approaches were used for food items not available in the USDA food composition database: (1) American terms were matched (i.e. the ECC is written in British English and the USDA database in American English). For example, “Courgette” was matched to zucchini; (2) if food items remained not found, we searched the internet for a description of the food item. For example, “Runner beans” were matched to kidney beans based on the information that kidney beans are the mature seed of the runner bean plant. For ECC pooled food items (e.g. salmon and trout), individual items were searched in the USDA database and the nutrient content was averaged.

The final nutrient intake was converted from weekly intake to daily intake (i.e. for each nutrient a nutrient in micrograms/day was created). If possible, portion sizes were adapted from individual studies, and otherwise based on USDA database information.

Person-years of follow-up for each participant was calculated from date of study enrolment until date of BC diagnosis, or date of ending follow-up (e.g. date of death, loss to follow-up, or study exit), whichever came first. For the NLCS study, a nested case–cohort approach was applied, in which the number of person-years at risk was estimated based on a sub-cohort that was randomly sampled [26].

Each study ascertained incident BC with International Classification of Diseases for Oncology (ICD-O-3 code C67) using population-based cancer registries, health insurance records, or medical records. The term BC was used for all urinary bladder neoplasms. BCs were classified into non-muscle invasive bladder cancer (NMIBC) and muscle invasive bladder cancer (MIBC). NMIBC included non-invasive papillary carcinomas confined to the urothelium (stage Ta), and carcinomas that invaded the lamina propria of the bladder wall (stage T1). High-grade flat non-invasive carcinomas confined to the urothelium (carcinoma in situ; CIS) without other concomitant tumour stages [i.e. T1/Ta (classified to non-muscle invasive prior) or MIBC] were also classified as NMIBC. MIBC included carcinomas that invaded into the detrusor muscle (stage T2), carcinomas that invaded into the peri-vesical tissue (stage T3), and carcinomas that invaded adjacent tissues and organs (most often the prostate or uterus, stage T4).

In addition to information on dietary intake, the BLEND data also included study characteristics (design, method of dietary assessment, recall time of dietary intake and geographical region), participant demographics (age, sex and ethnicity), BC pathology (MNIBC and MIBC), and smoking status (current/former/never and pack years), which were all measured at baseline [25].

### Statistical analysis

The differences between the different exposure categories were examined by *t* test for continuous variables. To assess the association between dietary B group vitamins and BC risk, cox proportional hazard regression analysis was used to obtain hazard ratios (HRs) and corresponding 95% confidence intervals, stratified by study centre. Scaled Schoenfeld residuals were estimated for each covariate to test the proportional hazards assumption. In addition, the appropriateness of the use of the log-normal distribution was tested using a Wald test, and again no evidence of violation was found [38].

Separate analyses were undertaken for each B group vitamin compound individually, for all B group vitamins combined, and for combinations of B group vitamins based on their biological function. Combining of B group vitamin intakes was done by multiplying the individual compound intakes. B group vitamin intake was classified in tertiles (low/moderate/high intake) based on the distribution of all included study participants per study separately, since not every study measured an equal amount of food items of focussed on the same food groups. The Cox-regression model used low consumers as the reference group and was adjusted for previously shown BC risk factors: model 1: adjusted for the confounders age, sex (male/female), smoking status (was defined as a dummy variable: 0 (never smokers); 1 [current light smokers (i.e. smoking less than 20 pack-years)]; 2 [current heavy smokers (i.e. smoking more than 20 pack-years)]; 3 [current smokers (no information on pack-years)]; 4 [former light smokers (i.e. smokers who ceased smoking over 1 year prior and smoked less than 20 pack-years)]; 5 [former heavy smokers (i.e. smokers who ceased smoking over 1 year prior and smoked more than 20 pack-years)]; 6 [former smokers (smokers who ceased smoking over 1 year prior and no information on pack-years)])) [5, 6] and model 2: additionally, adjusted for water (low/high) (because of the water-soluble origin of B group vitamins [23]).

The Wald-test was used to derive the *p* value for linear trend. To understand the relevance of the effect modification, the main interaction terms between B group vitamin consumption (low/moderate/high) and sex, smoking and water were added to the model. *P* interaction < 0.05 was considered statistically significant where upon all analyses were stratified for the covariate of interest. In addition, sensitivity analyses were performed in which BC cases diagnosed within the first 5 years after recruitment were excluded.

Based on a priori hypothesis, additional supplemental analyses were performed by BC subtype (i.e. NMIBC and MIBC). In addition, dose–response analyses of vitamin intake (plotted on the x-axis) and HR (model 2, plotted on the y-axis) were performed using a nonparametric test for trend.

All statistical analyses were performed using Stata14 (StataCorp LLC, College Station, TX).

## Results

### Baseline characteristics

Baseline characteristics of the study population are described in Table 1. In total, 3265 cases and 580,634 non-cases were included in our analyses. Cases were on average older than non-cases (60.3 vs 52.6 years), more likely to be current or former smoker (37.58% and 42.02% vs 20.82% and 29.15%, respectively) and there were approximately three times more male than female cases (2189 vs 726). Compared to non-cases, cases consumed on average more vitamin B1 (1.65 mg vs 1.01 mg), B2 (3.63 mg vs 0.97 mg), B3 (16.83 mg vs 12.11 mg), B6 (2.05 mg vs 1.51 mg) and B9 (282.81 µg vs 185.59 µg).

## B group vitamins and their associations with BC

### Overall results

Overall, no significant associations of higher intakes of any of the B group complex vitamins and BC risk were observed (Table 2), except for a moderate intake of vitamin B1, which showed to slightly increase the risk of BC (HR_B1_: 1.10, 95% CI: 1.00–1.20) (Table 2). No other significant associations were observed.

**Table 2:** Hazard ratios and 95% confidence intervals for BC according to dietary B group vitamin intake in the BLEND study

	Overall (*n* = 532,927)				Men (*n* = 171,771)				Women (*n* = 361,156)			
	Low	Moderate	High	*P*	Low	Moderate	High	*P*	Low	Moderate	High	*p*
Thiamin (B1)												
Model 1^a^	1	1.10 (1.00–1.20)	1.04 (0.95–1.14)	< 0.001	1	1.13 (1.02–1.26)	1.13 (1.02–1.26)	< 0.001	1	1.03 (0.87–1.22)	0.80 (0.67–0.97)	< 0.001
Model 2^b^	1	1.14 (1.03–1.26)	1.14 (1.01–1.29)	< 0.001	1	1.16 (1.03–1.30)	1.20 (1.05–1.38)	< 0.001	1	1.13 (0.94–1.36)	0.96 (0.75–1.23)	< 0.001
Riboflavin (B2)												
Model 1^a^	1	1.07 (0.98–1.17)	0.99 (0.91–1.08)	< 0.001	1	1.14 (1.02–1.26)	1.06 (0.95–1.17)	< 0.001	1	0.91 (0.76–1.08)	0.83 (0.70–1.00)	< 0.001
Model 2^b^	1	1.10 (1.00–1.21)	1.06 (0.93–1.21)	< 0.001	1	1.14 (1.02–1.28)	1.06 (0.91–1.23)	< 0.001	1	1.01 (0.83–1.22)	1.04 (0.80–1.35)	< 0.001
Niacin (B3)												
Model 1^a^	1	1.02 (0.93–1.12)	0.98 (0.90–1.07)	< 0.001	1	1.05 (0.94–1.17)	1.06 (0.95–1.18)	< 0.001	1	0.97 (0.82–1.15)	0.77 (0.63–0.93)	< 0.001
Model 2^b^	1	1.03 (0.94–1.14)	1.02 (0.91–1.14)	< 0.001	1	1.05 (0.94–1.18)	1.07 (0.94–1.21)	< 0.001	1	1.03 (0.86–1.24)	0.87 (0.68–1.10)	< 0.001
Pyridoxine (B6)												
Model 1^a^	1	1.00 (0.92–1.09)	0.95 (0.87–1.04)	< 0.001	1	1.05 (0.95–1.17)	1.05 (0.94–1.16)	< 0.001	1	0.89 (0.75–1.06)	0.73 (0.61–0.88)	< 0.001
Model 2^b^	1	1.01 (0.92–1.12)	0.99 (0.86–1.13)	< 0.001	1	1.06 (0.94–1.19)	1.06 (0.91–1.25)	< 0.001	1	0.92 (0.75–1.12)	0.79 (0.59–1.04)	< 0.001
Folate (B9)												
Model 1^a^	1	1.04 (0.95–1.14)	0.99 (0.90–1.08)	< 0.001	1	1.09 (0.98–1.21)	1.05 (0.95–1.17)	< 0.001	1	0.93 (0.79–1.11)	0.82 (0.68–0.99)	< 0.001
Model 2^b^	1	1.06 (0.96–1.16)	1.03 (0.93–1.14)	< 0.001	1	1.09 (0.98–1.22)	1.06 (0.94–1.20)	< 0.001	1	0.99 (0.83–1.19)	0.93 (0.75–1.16)	< 0.001
Cyanocobalamin (B12)												
Model 1^a^	1	0.97 (0.89–1.05)	0.94 (0.85–1.03)	< 0.001	1	0.96 (0.87–1.06)	1.01 (0.91–1.13)	< 0.001	1	0.99 (0.83–1.18)	0.79 (0.66–0.95)	< 0.001
Model 2^b^	1	0.97 (0.89–1.07)	0.95 (0.85–1.06)	< 0.001	1	0.95 (0.86–1.06)	1.00 (0.88–1.13)	< 0.001	1	1.04 (0.87–1.25)	0.88 (0.71–1.08)	< 0.001
Energy metabolism (B1*B2*B3)												
Model 1^a^	1	1.07 (0.98–1.17)	1.01 (0.92–1.10)	< 0.001	1	1.13 (1.02–1.26)	1.09 (0.98–1.21)	< 0.001	1	0.94 (0.79–1.12)	0.80 (0.66–0.96)	< 0.001
Model 2^b^	1	1.10 (1.00–1.22)	1.08 (0.96–1.23)	< 0.001	1	1.15 (1.02–1.28)	1.13 (0.98–1.30)	< 0.001	1	1.02 (0.84–1.24)	0.94 (0.73–1.21)	< 0.001
Oxidative stress reduction (B2*B6)												
Model 1^a^	1	1.02 (0.93–1.11)	0.96 (0.88–1.05)	< 0.001	1	1.08 (0.97–1.20)	1.05 (0.95–1.17)	< 0.001	1	0.89 (0.74–1.05)	0.74 (0.62–0.89)	< 0.001
Model 2^b^	1	1.04 (0.94–1.15)	1.01 (0.87–1.16)	< 0.001	1	1.08 (0.97–1.22)	1.08 (0.92–1.26)	< 0.001	1	0.92 (0.75–1.13)	0.80 (0.60–1.07)	< 0.001
DNA stability (B9*B12)												
Model 1^a^	1	1.00 (0.92–1.10)	0.95 (0.87–1.05)	< 0.001	1	1.01 (0.91–1.12)	1.04 (0.94–1.16)	< 0.001	1	0.99 (0.84–1.18)	0.76 (0.63–0.92)	< 0.001
Model 2^b^	1	1.02 (0.93–1.12)	0.99 (0.88–1.11)	< 0.001	1	1.02 (0.91–1.13)	1.06 (0.92–1.21)	< 0.001	1	1.04 (0.86–1.24)	0.84 (0.67–1.06)	< 0.001
Vitamin B complex (B1*B2*B3*B6*B9*B12)												
Model 1^a^	1	1.06 (0.97–1.15)	0.93 (0.84–1.03)	< 0.001	1	1.07 (0.97–1.18)	0.94 (0.84–1.06)	< 0.001	1	1.03 (0.86–1.24)	0.93 (0.77–1.11)	< 0.001
Model 2^b^	1	1.05 (0.96–1.14)	0.91 (0.82–1.01)	< 0.001	1	0.97 (0.97–1.18)	0.94 (0.83–1.06)	< 0.001	1	1.01 (0.84–1.20)	0.91 (0.76–1.09)	< 0.001

*BC* Bladder Cancer, *BLEND* BLadder cancer Epidemiology and Nutritional Determinants

^a^Adjusted for sex, age, and smoking status

^b^Adjusted for sex, age, smoking status and water

### Stratified analyses

#### Sex

A significant interaction was shown for sex and B vitamins (Table 3).

**Table 3:** *P* interaction values of possible confounders

	Thiamin (B1)	Riboflavin (B2)	Niacin (B3)	Pyridoxine (B6)	Folate (B9)	Cyanocobal amin (B12)	Energy metabolism (B1*B2*B3)	Oxidative stress reduction (B2*B6)	DNA stability (B9*B12)	Vitamin B complex (B1*B2*B3* B6*B9*B12)
Sex										
Moderate	0.33	0.03	0.44	0.11	0.13	0.64	0.07	0.06	0.96	0.29
High	0.001	0.02	0.003	0.001	0.02	0.02	0.003	0.001	0.004	0.002
Water	0.001	< 0.001	< 0.001	< 0.001	< 0.001	< 0.001	< 0.001	< 0.001	< 0.001	0.001
Moderate	0.96	0.87	0.09	0.40	0.76	0.66	0.61	0.39	0.58	0.83
High	0.59	0.41	0.53	0.70	0.80	0.42	0.41	0.68	0.35	0.181
Smoking										
Current light*moderate	0.48	0.65	0.16	0.73	0.44	0.71	0.33	0.14	0.94	0.40
Current light*high	0.16	0.15	0.13	0.39	0.31	0.91	0.20	0.43	0.88	0.21
Current heavy*moderate	0.23	0.91	0.91	0.09	0.88	0.56	0.82	0.68	0.41	0.69
Current heavy*high	0.88	0.88	0.60	0.96	0.14	0.02	0.92	0.82	0.02	0.93
Current unknown*moderate	0.05	0.21	0.29	0.83	0.50	0.29	0.16	0.16	0.90	0.05
Current unknown*high	0.04	0.17	0.03	0.25	0.53	0.95	0.09	0.10	0.78	0.20
Former light*moderate	0.79	0.75	0.71	0.85	0.57	0.19	0.59	0.55	0.08	0.73
Former light*high	0.60	0.77	0.67	0.98	0.52	0.74	0.71	0.82	0.86	0.47
Former heavy*moderate	0.69	0.95	0.82	0.54	0.63	0.40	0.87	0.54	0.94	0.02
Former heavy*high	0.31	0.23	0.70	0.17	0.26	0.31	0.32	0.20	0.46	0.21
Former unknown*moderate	0.57	0.64	0.23	0.84	0.28	0.29	0.28	0.93	0.41	0.82
Former unknown*high	0.15	0.23	0.12	0.30	0.54	0.26	0.18	0.14	0.37	0.11
BC type										
Moderate	0.56	0.81	0.46	0.24	0.04	0.73	0.87	0.76	0.63	0.72
High	0.30	0.78	0.32	0.95	0.65	0.95	0.52	0.41	0.25	0.33

Moderate intake of the vitamins B1, B2 and the vitamins related to energy metabolism showed to be associated with a small increased BC risk among men (HR_B1_: 1.13, 95% CI: 1.02–1.26, HR_B2_: 1.14, 95%CI: 1.02–1.26, HR_energy metabolism_: 1.13, 95% CI: 1.02–1.26). In addition, for vitamin B1 also high intake was related to an increased BC risk (HR_B1_: 1.13, 95% CI: 1.02–1.26) (Table 2).

No such positive associations were observed for women. In contrast, we observed an inverse associations between high intake of all B vitamins and B vitamin combinations and BC risk (HR_B1_: 0.80, 95% CI: 0.67–0.97, HR_B2_: 0.83, 95% CI: 0.70–1.00, HR_B3_: 0.77, 95% CI: 0.63–0.93, HR_B6_: 0.73, 95% CI: 0.61–0.88, HR_B9_: 0.82, 95% CI: 0.68–0.99, HR_B12_: 0.79, 95% CI: 0.66–0.95, HR_energy metabolism_: 0.80, 95% CI: 0.66–0.96, HR_oxidative stress_: 0.74, 95% CI: 0.62–0.89, HR_DNA stability_: 0.76, 95% CI: 0.63–0.92), except for the entire B group vitamin complex (Table 2).

### BC subtypes

#### MIBC

No significant interaction was shown for BC type and B vitamins (Table 3).

Overall, a decreased MIBC risk was observed for high intake of the vitamins B1, B2, B3, B6, B9 the vitamins related to energy metabolism, oxidative stress reduction and DNA stability (HR_B1_: 0.86, 95% CI: 0.77–0.97, HR_B2_: 0.88, 95% CI: 0.78–0.98, HR_B3_: 0.80, 95% CI: 0.72–0.90, HR_B6_: 0.88, 95% CI: 0.79–0.99, HR_B9_: 0.86, 95% CI: 0.77–0.96, HR_energy metabolism_: 0.84, 95% CI: 0.75–0.95, HR_oxidative stress_: 0.88, 95% CI: 0.79–0.98, HR_DNA stability_: 0.85, 95% CI: 0.75–0.95) (Table 4).

**Table 4:** Hazard ratios and 95% confidence intervals for MIBC according to dietary B group vitamin intake in the BLEND study

	Overall (*n* = 531,883)				Men (*n* = 170,954)				Women (*n* = 360,879)			
	Low	Moderate	High	*P*	Low	Moderate	High	*P*	Low	Moderate	High	*p*
Thiamin (B1)												
Model 1^a^	1	0.98 (0.87–1.09)	0.86 (0.77–0.97)	< 0.001	1	1.03 (0.90–1.17)	0.93 (0.82–1.06)	< 0.001	1	0.87 (0.70–1.08)	0.70 (0.55–0.88)	< 0.001
Model 2^2^	1	0.95 (0.84–1.07)	0.81 (0.70–0.95)	< 0.001	1	1.00 (0.87–1.15)	0.87 (0.73–1.04)	< 0.001	1	0.83 (0.65–1.06)	0.63 (0.46–0.87)	< 0.001
Riboflavin (B2)												
Model 1^a^	1	0.96 (0.86–1.08)	0.88 (0.78–0.98)	< 0.001	1	0.99 (0.87–1.13)	0.91 (0.80–1.03)	< 0.001	1	0.87 (0.70–1.08)	0.80 (0.63–1.00)	< 0.001
Model 2^2^	1	0.93 (0.82–1.05)	0.81 (0.69–0.96)	< 0.001	1	0.95 (0.82–1.10)	0.81 (0.67–0.98)	< 0.001	1	0.86 (0.67–1.11)	0.78 (0.56–1.09)	< 0.001
Niacin (B3)												
Model 1^1^	1	0.90 (0.81–1.01)	0.80 (0.72–0.90)	< 0.001	1	0.97 (0.85–1.10)	0.87 (0.77–1.00)	< 0.001	1	0.78 (0.63–0.97)	0.63 (0.50–0.82)	< 0.001
Model 2^2^	1	0.88 (0.78–0.99)	0.74 (0.64–0.86)	< 0.001	1	0.94 (0.82–1.08)	0.81 (0.69–0.95)	< 0.001	1	0.74 (0.58–0.93)	0.57 (0.42–0.77)	< 0.001
Pyridoxine (B6)												
Model 1^1^	1	0.89 (0.80–1.00)	0.88 (0.79–0.99)	< 0.001	1	0.94 (0.83–1.08)	0.96 (0.84–1.09)	< 0.001	1	0.79 (0.63–0.98)	0.71 (0.57–0.90)	< 0.001
Model 2^2^	1	0.86 (0.76–0.98)	0.80 (0.67–0.96)	< 0.001	1	0.92 (0.79–1.07)	0.90 (0.74–1.11)	< 0.001	1	0.70 (0.54–0.92)	0.57 (0.40–0.82)	< 0.001
Folate (B9)												
Model 1^1^	1	0.96 (0.86–1.08)	0.86 (0.77–0.96)	< 0.001	1	1.00 (0.88–1.14)	0.90 (0.78–1.02)	< 0.001	1	0.88 (0.71–1.10)	0.77 (0.60–0.98)	< 0.001
Model 2^2^	1	0.96 (0.85–1.08)	0.85 (0.74–0.97)	< 0.001	1	0.99 (0.86–1.14)	0.87 (0.75–1.02)	< 0.001	1	0.89 (0.70–1.11)	0.77 (0.58–1.02)	< 0.001
Cyanocobalamin (B12)												
Model 1^1^	1	0.93 (0.83–1.03)	0.90 (0.80–1.02)	< 0.001	1	0.93 (0.82–1.05)	0.96 (0.83–1.10)	< 0.001	1	0.93 (0.74–1.17)	0.82 (0.65–1.03)	< 0.001
Model 2^2^	1	0.93 (0.83–1.04)	0.90 (0.78–1.03)	< 0.001	1	0.92 (0.81–1.05)	0.94 (0.80–1.11)	< 0.001	1	0.94 (0.75–1.20)	0.85 (0.65–1.11)	< 0.001
Energy metabolism (B1*B2*B3)												
Model 1^1^	1	0.94 (0.84–1.06)	0.84 (0.75–0.95)	< 0.001	1	1.00 (0.87–1.14)	0.90 (0.79–1.03)	< 0.001	1	0.82 (0.66–1.02)	0.69 (0.55–0.88)	< 0.001
Model 2^2^	1	0.91 (0.80–1.03)	0.77 (0.66–0.90)	< 0.001	1	0.96 (0.83–1.11)	0.82 (0.68–0.98)	< 0.001	1	0.77 (0.60–0.98)	0.61 (0.44–0.85)	< 0.001
Oxidative stress reduction (B2*B6)												
Model 1^1^	1	0.89 (0.80–1.00)	0.88 (0.79–0.98)	< 0.001	1	0.92 (0.81–1.06)	0.94 (0.83–1.07)	< 0.001	1	0.82 (0.65–1.02)	0.73 (0.58–0.92)	< 0.001
Model 2^2^	1	0.86 (0.75–0.98)	0.80 (0.75–0.98)	< 0.001	1	0.90 (0.77–1.04)	0.87 (0.71–1.07)	< 0.001	1	0.74 (0.56–0.97)	0.60 (0.42–0.86)	< 0.001
DNA stability (B9*B12)												
Model 1^1^	1	0.89 (0.80–1.00)	0.85 (0.75–0.95)	< 0.001	1	0.90 (0.79–1.02)	0.90 (0.78–1.03)	< 0.001	1	0.90 (0.72–1.13)	0.76 (0.60–0.96)	< 0.001
Model 2^2^	1	0.88 (0.78–0.99)	0.82 (0.71–0.95)	< 0.001	1	0.88 (0.76–1.01)	0.86 (0.73–1.03)	< 0.001	1	0.89 (0.70–1.13)	0.73 (0.55–0.98)	< 0.001
Vitamin B complex (B1*B2*B3*B6*B9*B12)												
Model 1^1^	1	0.93 (0.83–1.04)	0.89 (0.79–1.01)	< 0.001	1	0.94 (0.83–1.07)	0.91 (0.78–1.05)	< 0.001	1	0.90 (0.71–1.13)	0.88 (0.70–1.10)	< 0.001
Model 2^2^	1	0.92 (0.82–1.02)	0.87 (0.76–0.99)	< 0.001	1	0.93 (0.82–1.06)	0.89 (0.76–1.04)	< 0.001	1	0.89 (0.70–1.12)	0.87 (0.69–1.09)	< 0.001

*MIBC* Muscle-Invasive Bladder Cancer, *BLEND* BLadder cancer Epidemiology and Nutritional Determinants

^1^Adjusted for sex, age, and smoking status

^2^Adjusted for sex, age, smoking status and water

Among men, a decreased MIBC risk was observed for high intake of vitamin B3 (HR_B3_: 0.87, 95% CI: 0.77–1.00) (Table 4).

Among women, a decreased MIBC risk was observed for moderate intake of vitamin B3 and B6 (HR_B3_: 0.78, 95% CI: 0.63–0.97, HR_B6_: 0.79, 95% CI: 0.63–0.98) and for high intakes of vitamin B1, B3, B6, B9, vitamins related to energy metabolism, oxidative stress reduction and DNA stability (HR_B1_: 0.70, 95% CI: 0.55–0.88, HR_B3_: 0.63, 95% CI: 0.50–0.82, HR_B6_: 0.71, 95% CI: 0.57–0.90, HR_B9_: 0.77, 95% CI: 0.60–0.98, HR_energy metabolism_: 0.69, 95% CI: 0.55–0.88, HR_oxidative stress_: 0.73, 95% CI: 0.589–0.92, HR_DNA stability_: 0.76, 95% CI: 0.60–0.96) (Table 4).

#### NMIBC

Overall, no significant association was observed between dietary vitamin B intake and NNMIBC risk (Table 5).

**Table 5:** Hazard ratios and 95% confidence intervals for NMIBC according to dietary B group vitamin intake in the BLEND study

	Overall (*n* = 532,271)				Men (*n* = 171,249)				Women (*n* = 361,022)			
	Low	Moderate	High	*P*	Low	Moderate	High	*P*	Low	Moderate	High	*p*
Thiamin (B1)												
Model 1^1^	1	1.06 (0.95–1.17)	1.02 (0.92–1.13)	< 0.001	1	1.09 (0.96–1.23)	1.10 (0.98–1.24)	< 0.001	1	1.00 (0.83–1.21)	0.79 (0.64–0.97)	< 0.001
Model 2^2^	1	1.10 (0.99–1.23)	1.12 (0.98–1.28)	< 0.001	1	1.11 (0.97–1.26)	1.17 (1.00–1.37)	< 0.001	1	1.11 (0.91–1.37)	0.98 (0.74–1.28)	< 0.001
Riboflavin (B2)												
Model 1^1^	1	1.03 (0.93–1.14)	0.96 (0.86–1.06)	< 0.001	1	1.09 (0.96–1.23)	1.02 (0.91–1.15)	< 0.001	1	0.89 (0.74–1.08)	0.80 (0.65–0.98)	< 0.001
Model 2^2^	1	1.05 (0.94–1.18)	1.01 (0.88–1.17)	< 0.001	1	1.08 (0.95–1.23)	1.01 (0.85–1.20)	< 0.001	1	1.00 (0.81–1.24)	1.02 (0.76–1.36)	< 0.001
Niacin (B3)												
Model 1^1^	1	0.97 (0.87–1.07)	0.95 (0.86–1.05)	< 0.001	1	0.99 (0.87–1.12)	1.03 (0.92–1.16)	< 0.001	1	0.94 (0.78–1.13)	0.73 (0.59–0.90)	< 0.001
Model 2^2^	1	0.98 (0.88–1.10)	0.99 (0.87–1.13)	< 0.001	1	0.99 (0.87–1.13)	1.04 (0.90–1.21)	< 0.001	1	1.00 (0.82–1.22)	0.83 (0.64–1.08)	< 0.001
Pyridoxine (B6)												
Model 1^1^	1	0.95 (0.85–1.05)	0.91 (0.82–1.00)	< 0.001	1	0.97 (0.86–1.09)	1.00 (0.89–1.12)	< 0.001	1	0.89 (0.74–1.08)	0.69 (0.56–0.84)	< 0.001
Model 2^2^	1	0.95 (0.85–1.06)	0.91 (0.78–1.07)	< 0.001	1	0.96 (0.84–1.10)	0.98 (0.81–1.18)	< 0.001	1	0.92 (0.74–1.15)	0.74 (0.54–1.01)	< 0.001
Folate (B9)												
Model 1^1^	1	1.02 (0.92–1.13)	0.97 (0.88–1.08)	< 0.001	1	1.07 (0.95–1.21)	1.04 (0.92–1.17)	< 0.001	1	0.90 (0.75–1.09)	0.79 (0.64–0.98)	< 0.001
Model 2^2^	1	1.04 (0.93–1.16)	1.02 (0.91–1.15)	< 0.001	1	1.08 (0.95–1.22)	1.06 (0.92–1.21)	< 0.001	1	0.97 (0.79–1.18)	0.91 (0.71–1.15)	< 0.001
Cyanocobalamin (B12)												
Model 1^1^	1	0.91 (0.83–1.00)	0.94 (0.84–1.04)	< 0.001	1	0.89 (0.80–1.00)	1.01 (0.89–1.15)	< 0.001	1	0.98 (0.80–1.19)	0.80 (0.65–0.97)	< 0.001
Model 2^2^	1	0.92 (0.83–1.02)	0.96 (0.85–1.09)	< 0.001	1	0.88 (0.79–1.00)	1.00 (0.86–1.15)	< 0.001	1	1.03 (0.84–1.26)	0.91 (0.72–1.15)	< 0.001
Energy metabolism (B1*B2*B3)												
Model 1^1^	1	1.04 (0.93–1.15)	0.98 (0.88–1.08)	< 0.001	1	1.09 (0.97–1.23)	1.06 (0.94–1.19)	< 0.001	1	0.92 (0.76–1.11)	0.77 (0.62–0.94)	< 0.001
Model 2^2^	1	1.07 (0.96–1.19)	1.05 (0.91–1.20)	< 0.001	1	1.10 (0.97–1.25)	1.09 (0.93–1.28)	< 0.001	1	1.01 (0.82–1.25)	0.93 (0.70–1.23)	< 0.001
Oxidative stress reduction (B2*B6)												
Model 1^1^	1	0.98 (0.89–1.09)	0.91 (0.83–1.01)	< 0.001	1	1.04 (0.92–1.17)	1.00 (0.89–1.13)	< 0.001	1	0.87 (0.72–1.05)	0.69 (0.57–0.85)	< 0.001
Model 2^2^	1	0.99 (0.88–1.11)	0.92 (0.79–1.08)	< 0.001	1	1.03 (0.90–1.17)	0.98 (0.82–1.18)	< 0.001	1	0.91 (0.72–1.13)	0.75 (0.55–1.03)	< 0.001
DNA stability (B9*B12)												
Model 1^1^	1	0.98 (0.89–1.08)	0.95 (0.85–1.05)	< 0.001	1	0.98 (0.87–1.10)	1.02 (0.90–1.16)	< 0.001	1	0.99 (0.82–1.20)	0.77 (0.63–0.95)	< 0.001
Model 2^2^	1	1.00 (0.90–1.11)	0.99 (0.87–1.13)	< 0.001	1	0.98 (0.87–1.11)	1.04 (0.89–1.21)	< 0.001	1	1.05 (0.86–1.28)	0.88 (0.69–1.14)	< 0.001
Vitamin B complex (B1*B2*B3*B6*B9*B12)												
Model 1^1^	1	1.01 (0.92–1.11)	0.92 (0.83–1.03)	< 0.001	1	1.03 (0.92–1.15)	0.93 (0.81–1.07)	< 0.001	1	0.95 (0.78–1.16)	0.90 (0.74–1.10)	< 0.001
Model 2^2^	1	0.99 (0.90–1.10)	0.90 (0.81–1.01)	< 0.001	1	1.03 (0.92–1.15)	0.92 (0.80–1.06)	< 0.001	1	0.93 (0.76–1.14)	0.91 (0.74–1.11)	< 0.001

*NMIBC* Non-Muscle Invasive Bladder Cancer, *BLEND* BLadder cancer Epidemiology and Nutritional Determinants

^1^Adjusted for sex, age, and smoking status

^2^Adjusted for sex, age, smoking status and water

Among men, moderate intake of vitamin B12 reduced the risk of NMIBC (HR_B12_: 0.89, 95% CI: 0.80–1.00) (Table 5).

Among women, high intake of the vitamins B1, B2, B3, B6, B9, B12 and vitamins related to energy metabolism, oxidative stress reduction and DNA stability decreased NMIBC risk (HR_B1_: 0.79, 95% CI: 0.64–0.97, HR_B2_: 0.80, 95% CI: 0.65–0.98, HR_B3_: 0.73, 95% CI: 0.59–0.90, HR_B6_: 0.69, 95% CI: 0.56–0.85, HR_B9_: 0.79, 95% CI: 0.64–0.98, HR_B12_: 0.80, 95% CI: 0.65–0.97, HR_Benergy metabolism_: 0.77, 95% CI: 0.62–0.94, HR_Boxidative stress_: 0.69, 95% CI: 0.57–0.85, HR_BDNA stability_: 0.77, 95% CI: 0.63–0.95) (Table 5).

### Removal of early BC cases

After removing BC cases diagnosed within the first 5 years after recruitment, similar increased BC risks were observed (Supplementary Table 1).

## Dose–response analyses

### Linear associations

**Fig. 1:**
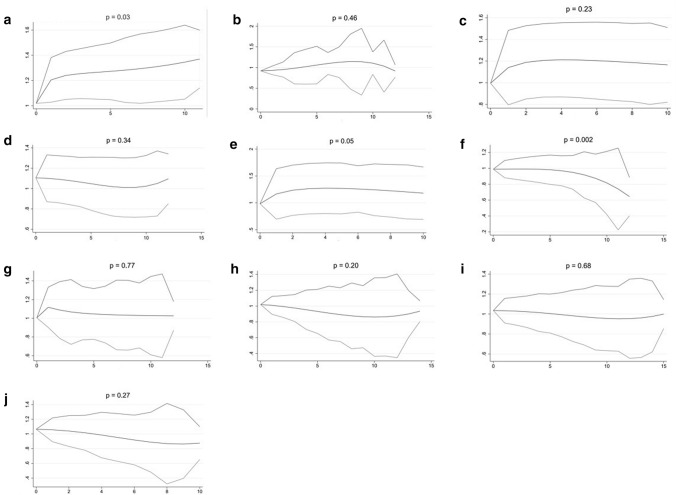
Dose–response analyses (x-axis: dose (defined per vitamin or vitamin group, see below), y-axis: HR for BC, with 95% confidence interval); **a** thiamin (B1) (dose = 300 µg), **b** riboflavin (B2) (dose = 800 µg), **c** niacin (B3) (dose = 2500 µg), **d** pyridoxine (B6) (dose = 300 µg), **e** folate (B9 (dose = 30 µg)), **f** cyanocobalamin (B12) (dose = 1.5 µg), **g** vitamins related to energy metabolism (dose = 1.25 × 10^11^ µg), **h** vitamins related to the reduction of oxidative stress (dose = 5.0 × 10^6^ µg), **i** vitamins related to DNA stability (dose = 200 µg) and **j** the entire B group vitamin complex (dose = 1.0 × 10^16^ µg)

Overall dose–response curves are shown in Fig. 1. A slightly increased BC risk was observed for higher intake of vitamin B1 (*p* = 0.03) and a decreased risk for higher intake of vitamin B12 (*p* = 0.002). No other compound or combination showed a significant association with BC risk.

**Fig. 2:**
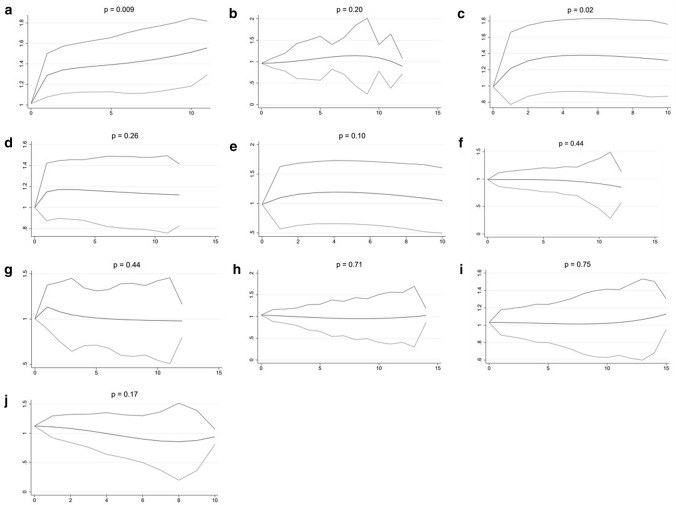
Dose–response analyses for men (x-axis: dose (defined per vitamin or vitamin group, see below), y-axis: HR for BC, with 95% confidence interval); **a** thiamin (B1) (dose = 300 µg), **b** riboflavin (B2) (dose = 800 µg), **c** niacin (B3) (dose = 2500 µg), **d** pyridoxine (B6) (dose = 300 µg), **e** Folate (B9 (dose = 30 µg)), **f** cyanocobalamin (B12) (dose = 1,5 µg), **g** vitamins related to energy metabolism (dose = 1.25 × 10^11^ µg), **h** vitamins related to the reduction of oxidative stress (dose = 5.0 × 10^6^ µg), **i** vitamins related to DNA stability (dose = 200 µg) and **j** the entire B group vitamin complex (dose = 1.0 × 10^16^ µg)

For men, a significant increased BC risk was observed for high intake of the vitamins B1 (*p* = 0.009) and B3 (*p* = 0.02) (Fig. 2).

**Fig. 3:**
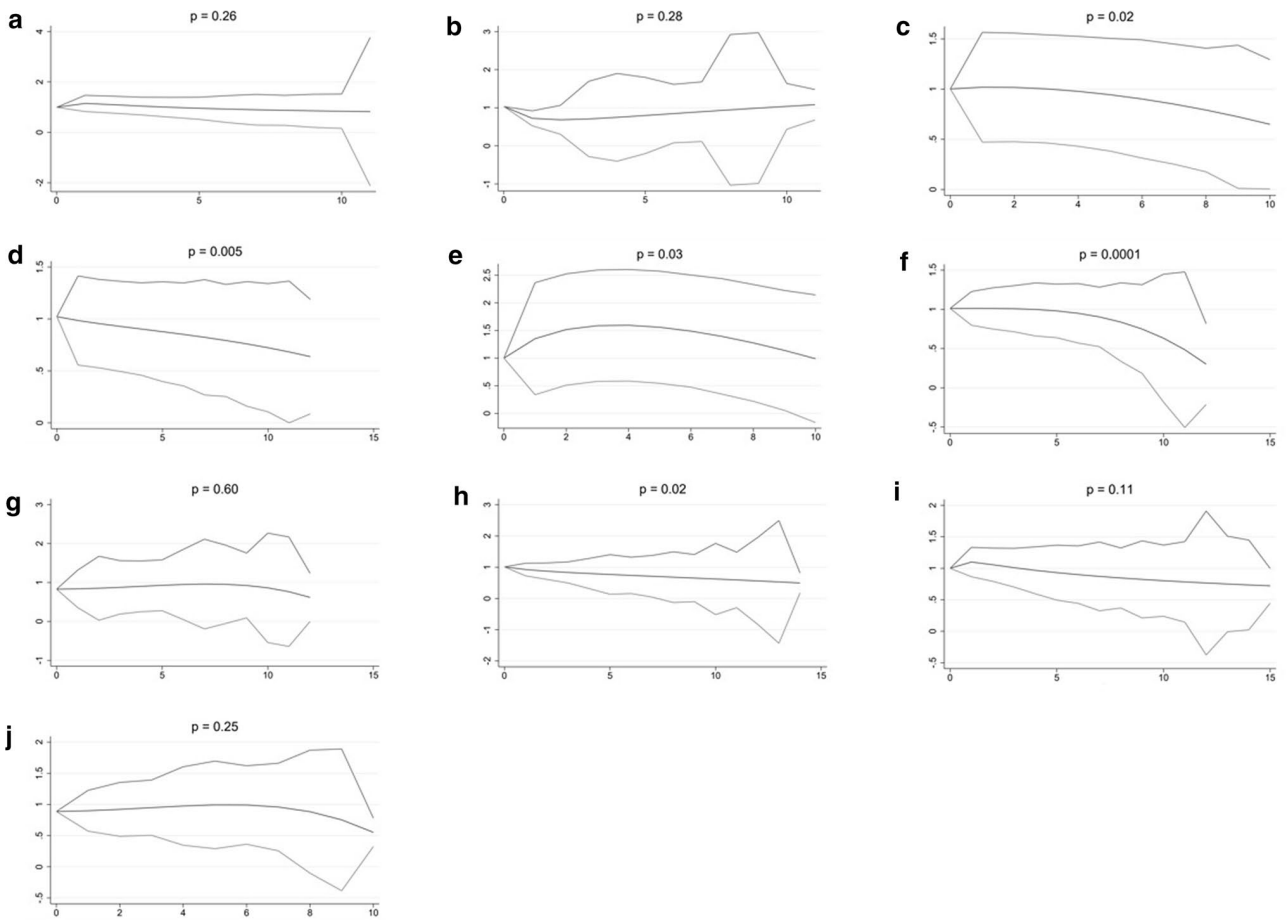
Dose–response analyses for women (x-axis: dose (defined per vitamin or vitamin group, see below), y-axis: HR for BC, with 95% confidence interval); **a** thiamin (B1) (dose = 300 µg), **b** riboflavin (B2) (dose = 800 µg), **c** niacin (B3) (dose = 2500 µg), **d** pyridoxine (B6) (dose = 300 µg), **e** folate (B9 (dose = 30 µg)), **f** cyanocobalamin (B12) (dose = 1.5 µg), **g** vitamins related to energy metabolism (dose = 1.25 × 10^11^ µg), **h** vitamins related to the reduction of oxidative stress (dose = 5.0 × 10^6^ µg), **i** vitamins related to DNA stability (dose = 200 µg) and **j** the entire B group vitamin complex (dose = 1.0 × 10^16^ µg)

Among women, significant decreased risks were observed for high intake of the vitamins B3 (*p* = 0.02), B6 (*p* = 0.005), B9 (*p* = 0.03), B12 (*p* = 0.0001) and for the vitamins related to the reduction of oxidative stress (*p* = 0.02) (Fig. 3).

## Discussion

The present study showed a slightly increased BC risk for moderate vitamin B1 consumption. In men, moderate intake of the vitamins B1, B2, and the vitamins related to energy metabolism and higher intake of vitamin B1 were associated with an increased BC risk, while in women, higher intake of all vitamins and vitamin combinations, except for the entire B group vitamin complex, showed an inverse association.

B group vitamins have essential roles in preventing transmission from a normal cell to a malignant cancer cell, by involving into the pathways of energy metabolism, oxidative stress reduction, and methylation regulation [39, 40].

### B group vitamins and energy metabolism

Experimental studies show that vitamin B1 is needed for the metabolism of glucose [41], thereby delivering the fuel of our cells [42]. Contrary, the present study showed a slight increased BC risk for moderate and high vitamin B1 intake among male, while among female high intake was associated with a decreased BC risk. No previous observational studies were conducted on the influence of vitamin B1 on BC risk, nonetheless, several attempts have been made to correlate vitamin B1 intake to other cancer types. However, results remain inconclusive [43, 44]. A possible explanation for the discrepant findings between male and female might be the different source from which participants retrieved their vitamin B1. While male mainly retrieved their vitamin B1 intake from meat consumption, the main vitamin B1 source for female are vegetables (Supplementary Table 2). Meat consumption has previously been associated with an increased BC risk [45–50], while high vegetable consumption showed a decreased BC risk [9, 10]. Moreover, after adjustment of the main food resources (i.e. meat and vegetables) results for men and women were similar and did not reach statistical significance, thereby strengthening the hypothesis that indeed the different food source might explain the observed gender difference. However, future research is needed to further clarify this gender difference.

Vitamin B2 is a coenzyme for many metabolic processes in the body [18, 41]. Although this suggests an inverse effect of vitamin B2 on BC risk, in current observational studies this effect is controversial [51]. The present study also shows conflicting results; among men moderate vitamin B2 intake showed an increased BC risk, while among women a decreased risk was observed. This gender difference might be explained by the source. It is expected that men mainly derive vitamin B2 from meat, while women might derive it mainly from other sources. This is confirmed by the higher vitamin B2 meat-derived intake in men compared to women (M_meat-B2 men_: 0.70 mg, SD = 0.63, M_meat-B2 women_: 0.55 mg, SD = 0.50, *t* = 90.96, *p* < 0.001) (Supplementary Table 2). Meat has previously been associated with an increased BC risk due to its pro-carcinogenic components [45–50], which could abolish the positive effect of vitamin B2. However, adjustment for the main food sources did not significantly change the findings. Therefore, future research is needed to clarify this gender difference.

Vitamin B3 plays a role in energy metabolism, which is essential to maintain cellular metabolism and respiration [52] and it is important for genetic and epigenetic regulators [52]. Studies of the consequences of DNA damage in cultured mouse and human cells as a function of vitamin B3 status have supported the hypothesis that vitamin B3 may be a protective factor in limiting carcinogenic events [53]. In line with this, we observed a decreased BC risk for high dietary vitamin B3 intake among women. Since meat is a rich source of vitamin B3 [54], this again could explain the observed gender differences. In our study, men retrieved on average more vitamin B3 from meat than women (M_meat-B3, men_: 17.83 mg, SD = 15.71, M_meat-B3 women_: 14.54 mg, SD = 13.44, *t* = 78.49, *p* < 0.001) (Supplementary Table 2). However, adjustment for the main food sources did not significantly change the findings. Therefore, future research is needed to clarify this gender difference. When analyzing the effect of the vitamins B1, B2 and B3 (i.e. the vitamins related to energy metabolism) together, moderate intake among men showed an increased risk on BC and high intake a decreased risk among women.

### B group vitamins and oxidative stress

Oxidative stress is an imbalance in the cell which leads to DNA damage [55], thereby increasing cancer risk [39]. Experimental studies showed that the vitamins B6 and B2 are involved in oxidative stress reduction by catalyzing regulatory enzymes [55, 56]. Results of the present study confirm these findings by showing an inverse association of high intake of vitamin B6 on BC risk among women, in line with a previously conducted cohort study [23].

### B group vitamins and DNA stability

Vitamin B9 plays a pivotal role in cell metabolism which is necessary for DNA synthesis and repair, as well as for methylation [57]. Previous epidemiological studies and a meta-analysis confirm this protective role of vitamin B9 in the development of BC [14]. The present study also shows a reduced BC risk among women (for both vitamin B9 separate as well as combined with vitamin B12). In men, this protective effect could not be observed. This is possibly the result of the fact that men are less responsive to vitamin B9 than women, due to a lower body mass in which the folate dose distributes over a larger volume [58]. The difference might also be explained by androgens, which are involved in one-carbon metabolism [59]. In our study, men did consume on average more vitamin B9 than women (M_B9, men_: 194.27 µg, SD = 128.66, M_B9 women_: 182.12 µg, SD = 96.28, *t* = 37.76, *p* < 0.001).

Vitamin B12 is essential for the reduction of DNA damage [60]. Experimental studies show that vitamin B12 deficiency mimics radiation damage to DNA, possibly leading to cancer [61]. However, observational studies showed no effect of high intake of vitamin B12 on BC risk [62]. The present study only observed a significant protective effect of high vitamin B12 intake among women. The main sources of vitamin B12 is meat [54]. Since meat is mainly consumed by men (M_B12, meat men_: 3.86 µg, SD = 4.30, M_B12 meat women_: 3.35 µg, SD = 3.85, *t* = 43.03, *p* < 0.001) (Supplementary Table 2), it might be argued that the observed effect in men are caused by the earlier mentioned negative effects of other (micro)nutrients in meat [63]. However, adjustment for the main food sources did not significantly change the findings. Therefore, future research is needed to clarify this gender difference.

### Limitations

Although BLEND is one of the largest known pooled cohort studies investigating the association between dietary B group vitamin intake and the risk of developing BC, allowing for detailed analyses with enough statistical power, it has several limitations. First, limited information was available for possible BC risk factors, such as body mass index, physical activity, socioeconomic status, and occupational exposures. Nevertheless, current literature shows only a small proportion of BC cases can be attributed to these factors [23, 64–66]. In addition, no information was available on comorbidities that may make people alter their diet [67], or of which the drugs may influence the bioavailability of B group vitamins in the body [68]. At last, information on alcohol was lacking which might influence B vitamins’ absorption [56].

A second limitation arises from the use of FFQs, which could lead to recall bias, systematic and random error when estimating vitamin intake. However, since the dietary intake of all included studies were validated, recall bias has likely only played a minor role. In addition, measurement error could be negligible, considering our large sample size.

Thirdly, although people are less likely to change their dietary habits at an older age, most of the included studies only measured their participants at baseline and we were, therefore, unable to take possible changes of dietary habits over time into account. This could have led to misclassification of long-term exposure [69]. However, the included NLCS study repeated the questionnaire 5 year after baseline, and showed only a minor decline in average intake for all food items [35].

Fourthly, most of the included studies did not provide information on supplement use. Therefore, we were unable to take supplemental vitamin intake into account, which may have led to an underestimation of the true effect of B group vitamins.

Fifthly, a single database was used for the conversion of food into nutrient intake. Since the food composition of similar food items and the food fortification may differ between different countries, the use of country specific food composition tables might be more accurate. Previous studies, however, showed that the use of a common food composition database advantages over the use of country-specific food composition databases in that errors are consistent between the countries [70]. In addition, all our main regression analyses were study centre stratified.

Besides, results obtained from cohort studies on diet and cancer risk cannot always rule out the possibility of reversed causality. Since there is no evidence that people are likely to alter their diet in the period before BC diagnosis, we decided to not exclude study participants who received a BC diagnosis within a short period of follow-up.

Finally, in view of multiple testing, it could indeed be debated whether, for instance, Bonferroni *p* value adjustments should have been applied. However, it previously has been argued that the use of Bonferroni *p* value adjustments is impractical and likely too conservative when testing a priori hypotheses [71]. Since we were able to formulate plausible a priori hypotheses regarding all the included analyses, based on data from previous studies, we did not apply Bonferroni correction in our analyses. Moreover, if we had adjusted for the number of main analyses being performed (*n* = 10) the significance level would have been 0.005. In that case, most of the observed associations between the vitamin B intake and BC risk would still be statistically significant*.*

## Conclusion

The present study showed a slight increased BC risk for moderate intake of vitamin B1. In men, moderate intake of the vitamins B1, B2 and the vitamins related to energy metabolism and high intake of vitamin B1 were found to be associated with an increased BC risk. In women, high intake of all vitamins and vitamin combinations, except for the entire complex, showed to have a protective effect. These findings may be helpful for informing BC prevention strategies. It should be noted, however, that dietary recommendations on vitamin B intake carefully consider the food sources from which this nutrient is retrieved. In addition, future studies should focus on nutritional patterns and look deeper into B group vitamins’ interactions with other nutrients.

## Supplementary Information

Below is the link to the electronic supplementary material.

Supplementary file1 (PDF 67 KB)

## Data Availability

The paper uses data of the BLEND study [25]. The data and code that support the findings of this study are available on reasonable request pending approval from the corresponding author, AW.
