# Supplementary material for: Dietary B group vitamin intake and the bladder cancer risk: a pooled analysis of prospective cohort studies
Source: Eur J Nutr. 2022 Feb 7;61(5):2397–416. doi: 10.1007/s00394-022-02805-2 (PMC9279207; doi:10.1007/s00394-022-02805-2)
Supplement: Supplementary file 1 — Supplementary file1 (PDF 67 KB) [file 394_2022_2805_MOESM1_ESM.pdf]

Supplementary Table 1*:* Hazard ratios and 95% confidence intervals for BC according to dietary B group vitamin intake in the BLEND study, excluding cases who received their diagnosis within 5 years after enrolment

	**Overall (n=532,231)**			
	**Low**	**Moderate**	**High**	**P**
**Thiamin (B1)**				
Overall	1	1.11 (1.00-1.23)	1.03 (0.93-1.15)	<0.001
**Riboflavin (B2)**				
Overall	1	1.07 (0.97-1.19)	0.96 (0.87-1.07)	<0.001
**Niacin (B3)**				
Overall	1	1.04 (0.94-1.15)	0.97 (0.87-1.07)	<0.001
**Pyridoxine (B6)**				
Overall	1	1.02 (0.92-1.13)	0.96 (0.87-1.07)	<0.001
**Folate (B9)**				
Overall	1	1.06 (0.96-1.18)	0.97 (0.87-1.08)	<0.001
**Cyanocobalamin (B12)**				
Overall	1	0.97 (0.88-1.07)	0.93 (0.83-1.04)	<0.001
**Energy metabolism (B1*B2*B3)**				
Overall	1	1.08 (0.98-1.20)	1.00 (0.90-1.11)	<0.001
**Oxidative stress reduction (B2*B6)**				
Overall	1	1.04 (0.94-1.16)	0.96 (0.87-1.06)	<0.001
**DNA stability (B9*B12)**				
Overall	1	1.01 (0.92-1.12)	0.93 (0.84-1.04)	<0.001
**Vitamin B complex (B1*B2*B3*B6*B9*B12)**				
Overall	1	1.10 (0.99-1.21)	0.95 (0.85-1.06)	<0.001

Supplementary Table 2*:* Average amount of consumed B group vitamins per week per food group per gender

Food source	Gender	Average B1 miligram (SD)	t (p)	Average B2 miligram (SD)	t (p)	Average B3 miligram (SD)	t (p)	Average B6 miligram (SD)	t (p)	Average B9 microgram (SD)	t (p)	Average B12 microgram (SD)	t (p)
Milk			77.62 (<0.001)		77.52 (<0.001)		72.80 (<0.001)		75.03 (<0.001)		0.01 (0.99)		32.57 (<0.001)
	Male	1.57 (7.96)		4.37 (20.97)		21.03 (117.50)		2.98 (15.93)		111.76 (266.31)		1.63 (13.66)	
	Female	0.39 (0.01)		1.18 (8.85)		4.83 (42.79)		0.71 (5.86)		111.75 (155.01)		0.67 (7.57)	
Egg			n.a.		n.a.		n.a.		n.a.		n.a.		n.a.
	Male												
	Female												
Meat			84.67 (<0.001)		90.96 (<0.001)		78.49 (<0.001)		73.36 (<0.001)		14.25 (<0.001)		43.03 (<0.001)
	Male	1.17 (1.22)		0.70 (0.63)		17.82 (15.71)		1.22 (1.10)		14.37 (22.17)		3.86 (4.30)	
	Female	0.91 (0.93)		0.55 (0.50)		14.54 (13.44)		1.01 (0.95)		13.58 (16.71)		3.35 (3.85)	
Fish			0.45 (0.65)		-0.97 (0.33)		-9.00 (<0.001)		-5.06 (<0.001)		-0.94 (0.35)		-4.51 (<0.001)
	Male	0.13 (0.19)		0.19 (0.25)		6.84 (7.77)		0.33 (0.38)		9.30 (12.11)		2.87 (3.33)	
	Female	0.13 (0.19)		0.19 (0.25)		7.39 (8.36)		0.35 (0.40)		9.38 (12.26)		2.98 (3.45)	
Grain			92.00 (<0.001)		92.97 (<0.001)		90.71 (<0.001)		76.85 (<0.001)		93.71 (<0.001)		118.41 (<0.001)
	Male	2.83 (2.19)		1.80 (1.38)		32.67 (25.34)		1.36 (1.11)		830.62 (625.70)		0.28 (0.22)	
	Female	2.33 (1.66)		1.48 (1.05)		26.98 (19.28)		1.15 (0.85)		683.56 (480.94)		0.22 (0.16)	
Nut			-84.57 (<0.001)		-34.94 (<0.001)		-18.69 (<0.001)		-76.17 (<0.001)		-44.03 (<0.001)		n.a.
	Male	0.17 (0.41)		0.11 (0.22)		1.84 (3.40)		0.17 (0.39)		26.91 (74.48)			
	Female	0.32 (0.68)		0.13 (0.27)		2.04 (3.87)		0.30 (0.67)		36.29 (71.07)			
Vegetables			-120.00 (<0.001)		-160.00 (<0.001)		-100.00 (<0.001)		-110.00 (<0.001)		-86.99 (<0.001)		-170.00 (<0.001)
	Male	1.78 (1.73)		1.18 (1.05)		19.55 (21.98)		4.33 (4.50)		269.47 (280.68)		17.73 (24.92)	
	Female	2.47 (2.05)		2.18 (2.41)		26.68 (23.54)		5.93 (5.07)		346.04 (306.25)		37.72 (46.45)	
Fruit			-44.40 (<0.001)		-17.47 (<0.001)		-3.80 (<0.001)		-11.09 (<0.001)		-28.95 (<0.001)		n.a.
	Male	0.10 (0.13)		0.20 (0.27)		1.76 (2.64)		0.77 (1.04)		45.39 (63.74)			
	Female	0.12 (0.12)		0.21 (0.26)		1.78 (2.54)		0.80 (1.05)		50.88 (64.52)			
Sugar			n.a.		n.a.		n.a.		n.a.		n.a.		n.a.
	Male												
	Female												

Beverages			23.34 (<0.001)		23.34 (<0.001)		23.34 (<0.001)		23.34 (<0.001)		23.78 (<0.001)	n.a.
	Male	0.33 (3.70)		0.48 (5.34)		1.66 (18.48)		0.77 (8.62)		37.65 (415.01)		
	Female	0.15 (1.49)		0.22 (2.16)		0.76 (0.74)		0.36 (3.48)		17.07 (166.53)		

Supplementary Table 2: Sensitivity analysis
